# Analysis of the *TID-I* and *TID-L* Splice Variants’ Expression Profile under In Vitro Differentiation of Human Mesenchymal Bone Marrow Cells into Osteoblasts

**DOI:** 10.3390/cells13121021

**Published:** 2024-06-11

**Authors:** Daniel Krakowian, Marta Lesiak, Aleksandra Auguściak-Duma, Joanna Witecka, Damian Kusz, Aleksander L. Sieroń, Katarzyna Gawron

**Affiliations:** 1 Department of Molecular Biology and Genetics, Faculty of Medical Sciences in Katowice, Medical University of Silesia, 40-055 Katowice, Poland; 2 Toxicology Research Group, Łukasiewicz Research Network—Institute of Industrial Organic Chemistry Branch Pszczyna, 43-200 Pszczyna, Poland; 3 Department of Parasitology, Faculty of Pharmaceutical Sciences in Sosnowiec, Medical University of Silesia, 41-200 Sosnowiec, Poland; 4 Department of Orthopaedics and Traumatology, Faculty of Medical Sciences in Katowice, Medical University of Silesia, 40-055 Katowice, Poland

**Keywords:** B-MSCs, osteogenic differentiation, TID/DNAJA3, alternative splicing variants, RNA silencing

## Abstract

Bone formation is a complex process regulated by a variety of pathways that are not yet fully understood. One of the proteins involved in multiple osteogenic pathways is TID (DNAJA3). The aim of this work was to study the association of TID with osteogenesis. Therefore, the expression profiles of the *TID* splice variants (*TID-L*, *TID-I*) and their protein products were analyzed during the proliferation and differentiation of bone marrow mesenchymal stromal cells (B-MSCs) into osteoblasts. As the reference, the hFOB1.19 cell line was used. The phenotype of B-MSCs was confirmed by the presence of CD73, CD90, and CD105 surface antigens on ~97% of cells. The osteoblast phenotype was confirmed by increased alkaline phosphatase activity, calcium deposition, and expression of ALPL and SPP1. The effect of silencing the *TID* gene on the expression of *ALPL* and *SPP1* was also investigated. The TID proteins and the expression of *TID* splice variants were detected. After differentiation, the expression of *TID-L* and *TID-I* increased 5-fold and 3.7-fold, respectively, while their silencing resulted in increased expression of *SPP1*. Three days after transfection, the expression of *SPP1* increased 7.6-fold and 5.6-fold in B-MSCs and differentiating cells, respectively. Our preliminary study demonstrated that the expression of *TID-L* and *TID-I* changes under differentiation of B-MSCs into osteoblasts and may influence the expression of *SPP1*. However, for better understanding the functional association of these results with the relevant osteogenic pathways, further studies are needed.

## 1. Introduction

Osteogenesis is a complex process requiring the interaction of numerous intracellular signaling pathways. Initially, mesenchymal stem cells differentiate into osteochondral progenitor cells that can further undergo osteogenesis or chondrogenesis. During embryogenesis in vivo, bone formation occurs through intramembranous ossification by osteoblasts or endochondral ossification by chondroblasts [1]. Differentiation of bone marrow mesenchymal stromal cells (B-MSCs) into osteoblasts under in vitro conditions consists of three stages that include cell proliferation, maturation of the extracellular matrix, and bone mineralization, [2] whilst the production and/or activity of characteristic proteins, such as osteopontin, osteocalcin, alkaline phosphatase, and Type I collagen have been reported [3,4,5]. For instance, alkaline phosphatase (EC:3.1.3.1) is involved in the mineralization and binding of collagen and calcium in bone, so increased activity of this enzyme is considered as an early marker of osteoblastic differentiation [6,7]. Otherwise, increased levels of osteocalcin and osteopontin are well known and established markers of mature osteoblasts [6]. Specific stages of B-MSCs’ differentiation can be distinguished by the expression of stem-cell-specific markers, such as those involved in cell adhesion and cell communication in mammalian cells, i.e., CD90 (Thy-1) [8], CD73 (5′NT—EC:3.1.3.5), [9] CD44, [10,11], or CD105 antigen (endoglin) [12].

An interesting group of proteins that participate in osteogenic differentiation are the heat shock proteins (HSPs), for which increased expression of HSP27/HSPB and HSP90/HSPC or a decrease in HSP70/HSPA have been demonstrated [13,14]. Specifically, the latter one forms complexes with DNAJ proteins, which are crucial for cells’ growth and survival, and participates in the assembly, folding, and unfolding of a number of secreted proteins; in their translocation across cell membranes; and prevents their aggregation [15,16,17,18]. One of the DNAJ family proteins is represented by tumorous imaginal disc (TID), also known as DNAJA3 (DnaJ homolog subfamily A member 3, mitochondrial), whose physical and functional interactions with HSPA proteins have been clearly demonstrated [15,17,19]. In addition, the expression of *TID-I* was shown to inhibit the phosphorylation of HSP27 [20]. Moreover, TID proteins have been linked to multiple pathways and proteins that may regulate osteogenesis, e.g., WNT [21], Hedgehog pathways [22], Janus Kinase/Signal Transducer and Activator of Transcription [23,24,25,26], Mothers Against Decapentaplegic Homolog family proteins [27], Epidermal Growth Factor Receptor [28,29], Hepatocyte Growth Factor Receptor [30], Neurotrophic Receptor Tyrosine Kinase [31], and Muscle, Skeletal Receptor Tyrosine-Protein Kinase [32,33]. In addition, the expression of *TID* induces autophagy [34], which regulates the regenerative function of B-MSCs and contributes to the development of osteoporosis [35].

*TID* is expressed in a developmental stage- and/or in a cell-type-specific manner [16,21]. Its expression has been detected in almost all organs and tissues, including bone marrow and bones [21,36]. It is also known as a tumor suppressor that regulates viral oncoproteins [37].

The original transcript of the *TID* gene is alternatively spliced to produce three variants, namely *TID-L*, with the complete sequence of Exons 1 to 11; *TID-I*, with a spliced sequence between Exon 10 and Exon 12; and *TID-S*, with 150 nucleotides removed in Exon 5 [38]. Additionally, these transcript variants serve for the production of six proteins, including three mitochondrial proteins, i.e., TID-Lm, TID-Im, and TID-Sm, and three cytoplasmic proteins, i.e., TID-Lc, TID-Ic, and TID-Sc [21]. One variant may dominate over the others in different tissues [38].

TID proteins play key roles in numerous pathways and processes that are crucial for stem cells’ differentiation into osteoblastic cells [21,37], as well as the proliferation and differentiation of other types of cells [37]. However, the impact of specific *TID* splice variants on osteogenesis has not been studied so far.

To verify it, here, we specifically analyzed the expression profiles and protein products of the *TID-L* and *TID-I* variants during the proliferation and differentiation of human B-MSCs into osteoblasts. 

## 2. Materials and Methods

### 2.1. Isolation and Culture of B-MSCs

Human B-MSCs were collected from a healthy adult bone marrow donor after orthopedic surgery, as described elsewhere [39]. Briefly, fragmented bone marrow tissue was suspended in alpha-Minimum Essential Medium (α-MEM) containing 10% fetal bovine serum (FBS), L-glutamine, and an antibiotic–antimycotic solution. The resulting cell suspension was inoculated into a culture flask and incubated overnight at 37 °C in 5% CO_2_. The next day, the culture medium was replaced, and the adherent cells were cultured until 90% confluence. Cells were then trypsinized, frozen in α-MEM containing 20% FBS and 10% dimethyl sulfoxide, and stored in liquid nitrogen for the experiments. Prior to the experiments, the cells were thawed and cultured in MesenCult™ XF medium for B-MSCs. 

The study was carried out in accordance with the Declaration of Helsinki and was approved by the Bioethics Committee of the Silesian Medical University in Katowice, Poland (KNW/0022/KB1/56/I/109). Prior to the study, written informed consent was obtained from the donor of the B-MSCs.

### 2.2. Proliferation of B-MSCs

B-MSCs were plated in MesenCult™XF Basal Medium (Stem Cell Technologies, Vancouver, BC, Canada) supplemented with 20% MesenCult™XF Supplement, 2 mM L-glutamine (PAA Laboratories GmbH, Pasching, Austria) and a 1% antibiotic and antimycotic solution (PAA Laboratories GmbH, Pasching, Austria) containing penicillin (10.000 U/mL), streptomycin sulphate (10 mg/mL), amphotericin B (25 μg/mL), and sodium chloride (0.9%) in the culture vessel coated with MesenCult™SF Attachment Substrate (Stem Cell Technologies, Vancouver, BC, Canada) with phosphate-buffered saline (PBS) at a ratio of 1:28. Cells were cultured under standard conditions (37 °C, 5% CO_2_) until confluence and used for experiments at the fifth passage. To split the cells, a MesenCult™ ACF Dissociation Kit (Stem Cell Technologies, Vancouver, BC, Canada) was used.

### 2.3. Differentiation of B-MSCs

B-MSCs were seeded into the culture vessel at a density of ~1.08 × 10^4^/cm^2^ and incubated for 24 h, as described above (MesenCult™ XF). Subsequently, the medium was replaced with the MesenCult™ Osteogenic Stimulatory Kit (Human) (Stem Cell Technologies, Vancouver, BC, Canada), and the culture continued without passaging for 28 days. The kit contained the MesenCult™ MSC Basal Medium with supplements (osteogenic stimulatory supplement, 15%; 3.5 mM β-glycerophosphate, 10^−8^ M dexamethasone, and 50 μg/mL ascorbic acid; Stem Cell Technologies, Vancouver, BC, Canada), 2 mM L-glutamine (PAA Laboratories GmbH, Pasching, Austria), and a 1% antibiotic–antimycotic solution (PAA Laboratories GmbH, Pasching, Austria). Cells were incubated under standard culture conditions (37 °C, 5% CO_2_) until the experiments were performed.

### 2.4. Culture of Control Cells

As a control, a commercially available immortalized immature human pre-osteoblast line, hFOB1.19, transfected with the heat-sensitive large T antigen of SV40 virus (ATCC^®^ CRL-11372™), was used. When cultured at 39.5 °C, the proliferation of hFOB1.19 is inhibited, and the cells express a normal osteoblast phenotype [40]. Additionally, similar to B-MSCs, the hFOB1.19 cells can be differentiated into other cell types, e.g., adipocytes or chondrocytes [41], and they express identical cell surface markers [42].

In all experiments, the hFOB1.19 cell line before the fourth passage was used as a control. Cells were cultured in Dulbecco’s Modified Eagle Medium/Nutrient Mixture F-12 without phenol red (Gibco, Waltham, MA, USA), supplemented with 10% FBS (PAN Biotech GmbH, Aidenbach, Germany), a 1% antibiotic–antimycotic solution (PAA Laboratories GmbH, Pasching, Austria), 2 mM L-glutamine (PAA Laboratories GmbH, Pasching, Austria), and 0.3 mg/mL G 418 (Sigma Aldrich, St. Louis, MO, USA). Prior to the study, the cells were grown at 37 °C and passaged at subconfluence using 0.05% trypsin and 0.02% ethylenediaminetetraacetic acid (PAN Biotech GmbH, Aidenbach, Germany) in PBS. Immediately prior to the study, the cells were plated at a density of ~1.08 × 10^3^/cm^2^. After 24 h of incubation, the cells were transferred to 39.5 °C and cultured continuously for 10 days without further passages to identify osteogenic markers [40,41,42,43,44].

### 2.5. Analysis of Cell Proliferation 

To determine cell proliferation, 2 × 10^4^ cells (B-MSCs or hFOB1.19) per well were seeded in a 24-well plate (1.85 cm^2^) and cultured with 10% Alamar Blue (Life Technologies, Waltham, MA, USA) for 2 h. Fluorescence measurement of resorufin was then performed using a Victor-X5 plate reader (Perkin-Elmer, Waltham, MA, USA). Simultaneously, growth curves were generated for B-MSCs and hFOB1.19 cells (R^2^ > 0.9950), and the population’s doubling time corresponding to 72 h of culture was calculated for all the cells examined. The number of cells was determined after 1, 2, 3, and 5 days of culture for B-MSCs under conditions of proliferation, and after 1, 2, 3, 4, 5, 7, 14, and 28 days for conditions of differentiation. The hFOB1.19 cells were cultured at 39.5 °C and 37 °C for 1, 2, 3 and 7 days for a comparison of proliferation (Table 1). Media with 10% Alamar Blue were used as a blank. The proliferation rate was observed visually as the change in cell numbers between consecutive days of analysis (i.e., the growth of cells in 1 day).

### 2.6. Flow Cytometry

Analysis of the cells’ phenotype was performed using B-MSCs cultured under proliferation and differentiation conditions after 4, 7, 14, and 28 days of culture, as well as, the hFOB1.19 cell line at 39.5 °C for 10 days of culture and at 37 °C to check the population differences, according to the criteria adopted for B-MSCs [45]. Briefly, B-MSC markers (CD90, CD105, CD73), hematopoietic stem cell markers (CD34, CD11b, CD19, CD45, HLA-DR), and CD44 antigens were detected using the BD Stemflow™ Human MSC Analysis Kit (BD Biosciences, Franklin Lakes, NJ, USA). After detachment and centrifugation (250× *g* for 5 min), the cells were resuspended in PBS. To recover the cells after 7, 14, and 28 days of differentiation, additional digestion of the extracellular matrix was performed using 2 mg/mL (284 u/mg) of Type I collagenase (Gibco, Waltham, MA, USA) and 0.5% trypsin for 1 h at 37 °C. The cells were incubated in the dark for 40 min at RT using antihuman antibodies (CD90-FITC, CD105-PerCP-Cy5.5, CD73-APC, CD44), a negative isotype control (mIgG1, κ PE, mIgG2a, κ PE), a positive isotype control (mIgG1, ĸFITC, mIgG1, ĸPerCp-Cy5.5, mgIG1, ĸAPC), a positive cocktail (CD90-FITC, CD105-PerCP-Cy5.5, CD73-APC), and a negative cocktail (CD34-PE, CD11b-PE, CD19-PE, CD45-PE and HLA-DR-PE). After centrifugation, the unbound antibodies were removed and the labeled cells were resuspended in a wash buffer and analyzed using a FACS Aria I flow cytometer (BD Biosciences, Franklin Lakes, NJ, USA).

### 2.7. Alkaline Phosphatase Activity Assay

The cells were fixed with 4% paraformaldehyde in 0.1 M PBS (pH 7.3) for 20 min at RT with shaking. After washing with PBS and deionized water, the 5-bromo-4-chloro-3-indolyl phosphate and nitro tetrazolium blue solution (Sigma-Aldrich, St. Louis, MO, USA) was added to the wells and left in the dark for 20 min. The reaction was stopped with deionized water, and stained cells were observed by light microscopy under phase contrast (Olympus IX71, Olympus, Shinjuku City, Japan).

Quantification of the alkaline phosphatase activity was performed in a separate experiment (SIGMAFAST p-Nitrophenyl Phosphate Tablets, Sigma-Aldrich, St. Louis, MO, USA). Cultured cells were rinsed twice with water and incubated with pNPP in a 0.2 M Tris buffer for 5 min at RT. The reaction was stopped by adding 3 N NaOH (1 part of NaOH per 4 parts of the pNPP solution), and absorbance was measured at 405 nm using Victor-X5 plate reader (PerkinElmer, Waltham, MA, USA). The results were referenced to the number of cells determined by the Alamar Blue assay. The amount of enzyme was calculated using a standard curve for alkaline phosphatase (FastAP; Fermentas, Waltham, MA, USA) (R^2^ = 0.9990). In addition, the rate of the increase in alkaline phosphatase per day between successive days of differentiation was calculated for B-MSCs differentiating into osteoblasts.

### 2.8. Alizarin Red Staining

Mineralization nodules were detected in B-MSCs cultured for 5 days (proliferation) and differentiating for 4, 7, 14, and 28 days according to a previously described procedure. As controls, hFOB1.19 cells cultured for 10 days were used [46]. The cells were fixed using 70% ice-cold ethanol and kept at −20 °C overnight. Next day, the cells were dehydrated with 96% ethanol, rinsed with deionized water, and incubated with a 40 mM Alizarin Red solution (Alizarin Red-S, Sigma-Aldrich, St. Louis, MO, USA; pH 4.2) for 10 min at RT on a shaker. After rinsing and removal of unspecific binding (PBS, 15 min), red calcium inclusions were observed under a phase-contrast microscope (Olympus IX71 light microscope, Olympus, Shinjuku City, Japan) and analyzed using Cell^F version 3.2 software.

Next, the cells were incubated in a desorbing solution, i.e., 10% cetylpyridinium chloride (Sigma-Aldrich, St. Louis, MO, USA), in a 10 mM sodium phosphate buffer (pH 7.0) for 15 min at RT. The resulting solution was diluted 10-fold, and the absorbance was measured at 562 nm. The results were compared with the standard curve for Alizarin, dissolved at different concentrations in cetylpyridinium chloride to calculate the amount of calcium deposits, considering that 1 mol of Alizarin binds 2 mol of calcium (R^2^ = 0.9977) (Supplementary Materials). The results were referenced to the number cells determined by the Alamar Blue assay and the number of cells per well on the day of culture. In addition, the rate of the increase in calcium between successive days of differentiation (per day) was calculated for B-MSCs differentiating into osteoblasts.

### 2.9. Western Blotting

In the assay, proliferating (5 days of culture) and differentiating (4, 7, 14, and 28 days of culture) B-MSCs as well as hFOB1.19 cells as controls (cultured for 10 days) were used. The cells were treated with a lysis buffer (150 mM NaCl, 1% Nonidet P40, 0.5% sodium deoxycholate, 50 mM Tris; pH 7.5; Roche, Basel, Switzerland) supplemented with protease inhibitors (Complete Tablet, Roche, Basel, Switzerland).

The cell suspensions were collected and homogenized on ice for 10 min. The homogenate was centrifuged at 13,000× *g* for 15 min at 4 °C, and the sample was frozen at −80 °C. The protein concentrations were determined by the Bradford method. In brief, Coomassie (Bradford) and a Protein Assay Kit (Thermo Scientific, Waltham, MA, USA) were used, and they absorbance was measured at 595 nm using an Eppendorff BioPhotometer plus a spectrophotometer (Eppendorf AG, Hamburg, Germany). A standard curve for bovine serum albumin (BSA) concentrations was generated (R^2^ = 0.9966) (Supplementary Materials).

Next, 4 µg of the protein sample was denatured at 95 °C for 5 min and loaded onto 10% polyacrylamide gels. The electrophoresis was carried out in a TGS buffer (Bio-Rad, Hercules, CA, USA) containing 192 mM glycine, 0.1% sodium dodecyl sulfate, and 25 mM Tris (pH 8.3). A direct current voltage of 100 V was used initially, which was increased to 120 V after 10 min of separation and followed by electrotransfer onto polyvinylidene difluoride (Immobilon-P Transfer Membrane, Millipore, Burlington, VT, USA) in a TGS buffer containing 20% methanol for 90 min at 500 mA. The membrane was incubated for 1 h in a TBST buffer (150 mM NaCl, 10 mM Tris, 0.05% Triton-X100; pH 7.6) with Anti-Actin (20-33) antibody produced in rabbit (1.35 ng/mL) (Sigma-Aldrich, St. Louis, MO, USA) or 2 μg/mL of rabbit polyclonal anti-TID antibody (E3) [21,22]. After washing, the membranes were incubated with horseradish peroxidase-conjugated secondary antibody (~300 pg/mL; Sigma-Aldrich, St. Louis, MO, USA) for 30 min, followed by the final incubation in Quantum reagent (Advansta, San Jose, CA, USA).

### 2.10. Real Time q-PCR

The RNA purification procedure was performed using TRIzol Reagent (Invitrogen, Waltham, MA, USA). The cell lysates thus obtained were frozen at −80 °C for further steps according to the TRIzol Reagent User Guide. The air-dried RNA was suspended in RNase-free water for evaluations of the concentration and quality (Nano-drop 2000 spectrophotometer; Thermo Scientific, Waltham, MA, USA).

The cDNA was prepared according to the manufacturer’s protocol using the SuperScript II Reverse Transcriptase Kit (Invitrogen, Waltham, MA, USA), 500 μg/mL oligo(dT)_12-18_ (Sigma-Aldrich, St. Louis, MO, USA), 10 mM dNTP mixture (Sigma-Aldrich, St. Louis, MO, USA), and 2 μg of total RNA. Prior to the reverse transcription reaction, DNA was removed using the TURBO DNA-free Kit (Ambion, Waltham, MA, USA).

Twenty nanograms of total RNA transcribed into cDNA was used for RT-qPCR. LightCycler 480 SYBR Green and Master Mix reagent (Roche, Basel, Switzerland) were used in the Light Cycler 480II (Roche, Basel, Switzerland) and QuantiTect SYBR Green PCR Kits (Qiagen, Venlo, The Netherlands) that were used in an ABI PRISM 7300HT (Applied Biosystems, Waltham, MA, USA). The reaction in the Light Cycler 480II was as follows: 8 min of initial denaturation at 95 °C, followed by 45 cycles of 13 s at 95 °C, 5 s at 60 °C, 35 s at 72 °C, 2 s at 80 °C (removal of unspecific signals and reading of fluorescence). The reaction in the ABI PRISM 7300HT was as follows: 15 min of initial denaturation at 95 °C, followed by 40 cycles for 30 s at 95 °C, 30 s at 54 °C, and 60 s at 72 °C (reading of fluorescence). At the end of the reaction, a melting curve was checked for each product. Next, 5 pM of the primers for the *GAPDH*, *SPP1*, and *ALPL* genes or 10 pM of the primers for the coding sequences of *TID-L*, *TID-I*, *TID-S*, and *HPRT1* were applied. The sequences of the primers used in the study [47,48] are included in Table 2. The efficiency of PCR for primers was determined with 10-fold dilutions of the starting cDNA (0.2–200 ng of total RNA). The yield was determined using an equation (Supplementary Materials). The efficiency of the PCR was over 90% for all genes, and the results were analyzed via the 2^−ΔΔCt^ method [49]. The change in the expression was determined by comparison with the reference genes (*GAPDH* or *HPRT*). Relative expression (ΔCt) was compared with the relative gene expression in B-MSCs (2^−ΔΔCt^) and analyzed using LibreOffice 4.2 software.

### 2.11. Silencing of Gene Expression by siRNA

Silencing of the expression of the *TID* gene was carried out using commercially available synthetic siRNA sequences, i.e., DNAJA3_8 (CCG GAT TAA CAG CTA CGG CTA), DNAJA3_5 (CTC CGG CAT GGA AAC CAT CAA), DNAJA3_7 (CCC GAG CGC TGC TGA CAT TGA), and DNAJA3_9 (AAA GGC CAT GCT TAC AGC TTA) (Qiagen, The Netherlands) (Supplementary Materials). The control siRNA (AllStars Negative Control siRNA, Qiagen, Venlo, The Netherlands) was used as a reference (negative control) to prevent any non-specific silencing effect. An untransfected control was used to check the effect of the transfection on the cells. The AllStars HS Cell Death Control siRNA (Qiagen, Venlo, The Netherlands), which induces cell death after transfection, was used as a control. Prior to the study, the transfection protocol was optimized. The B-MSCs were seeded into 24-well plates at a concentration of 3.2 × 10^4^ cells per well. After 2 h of incubation at 37 °C, 10 nM of siRNAs and 4.5 µL of HiPerFect (Qiagen, Venlo, The Netherlands) were added to the cell cultures. Further, the cells were incubated at 37 °C for 24, 48, 72, and 96 h under conditions of differentiation and for 24, 48, and 72 h under conditions of proliferation. Finally, the efficiency of silencing was calculated using the 2^−ΔΔCt^ method [49]; the relative expression of *TID-L* and *TID-I* was compared with the negative control. The siRNA used for analysis was the one that produced the optimal level of silencing under conditions of either proliferation or differentiation.

Both human B-MSCs and cells differentiating into osteoblasts were detached and resuspended in a lysis buffer (150 mM NaCl, 1 mM ethylenediaminetetraacetic acid, 1% NP-40, 5% glycerol, 25 mM Tris; pH 7.4) (Pierce, Waltham, MA, USA), supplemented with protease and phosphatase inhibitors (Halt Protease & Phosphatase Inhibitor Cocktail; Pierce, Waltham, MA, USA). Then 3 μg of proteins was applied and analyzed by Western blotting, as previously described. The proliferation assay and the alkaline phosphatase activity were performed (as described above) on B-MSCs cultured for 2 days (proliferation) as well as for 3, 5, and 14 days (differentiation) and used for the detection of calcium deposits.

### 2.12. Statistical Analysis

The data are presented as the means ± standard deviation. Statistical tests of the results (proliferation, flow cytometry, alkaline phosphatase activity, calcium deposits, real-time q-PCR) were performed using Student’s *t*-test in comparison with the corresponding results obtained for the undifferentiated B-MSCs. In the case of the silencing of *TID* genes’ expression, statistical significance was calculated against the negative control of transfection using Student’s *t*-test. The results were considered statistically significant at *p* < 0.05. The data were analyzed using LibreOffice 4.2 software.

## 3. Results

### 3.1. The Proliferation Rate of B-MSCs Decreases under Conditions of Differentiation

**Figure 1:**
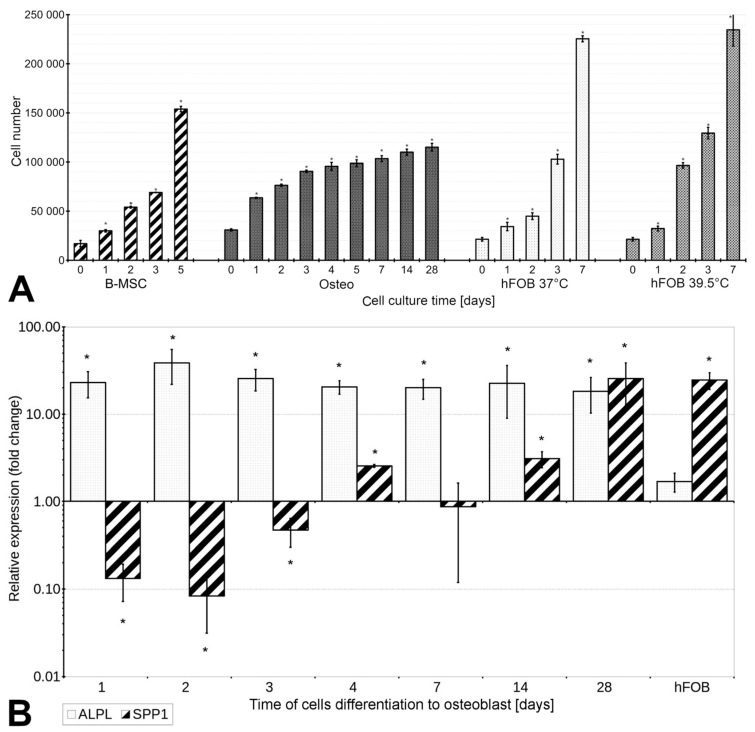
Cells’ proliferation and expression of osteogenic markers in B-MSCs differentiating to osteoblasts. (**A**) The proliferation was analyzed with the use of metabolic Alamar Blue assays. The number of cells was determined based on the fluorescence values compared with the standard curves for B-MSCs and hFOB1.19 cells (*n* = 4). B-MSCs, bone marrow mesenchymal stromal cells; Osteo, B-MSC differentiating to osteoblasts; hFOB 37 °C, hFOB1.19 cultured at 37 °C; hFOB 39.5 °C, hFOB1.19 cultured at 39.5 °C; d, day of culture. Each analysis was repeated in quadruplicate. * The results were considered statistically significant at *p* < 0.05. (**B**) Relative expression of alkaline phosphatase (*ALPL*) (little grey dots) and osteopontin (*SPP1*) (black diagonal stripes) during the differentiation of B-MSCs to osteoblasts for 4, 7, 14, and 28 days and in hFOB1.19 (hFOB) cells cultured for 10 days at 39.5 °C, compared with the expression in undifferentiated B-MSCs. The experiments were performed in quadruplicate. * The results were considered statistically significant at *p* < 0.05.

The population-doubling time of B-MSCs was approximately 35 h under conditions of proliferation and 46 h under conditions of differentiation. For both tested conditions, the increase in cell numbers compared with Day 0 was statistically significant (*p* < 0.05). The proliferation rate of B-MSCs was constant under conditions of proliferation (a steady increase in the number of cells from one day of culture to another), but varied under conditions of differentiation, when the number of cells doubled after the first day of differentiation, and then the proliferation rate gradually decreased but did not stop completely until Day 28 (Figure 1A). Therefore, the B-MSCs cultured under differentiating conditions showed a lower proliferation rate compared with undifferentiated cells.

The proliferation rate of hFOB1.19 cells increased in comparison with B-MSCs at both, 39.5 °C and 37 °C (the population-doubling times were 32 h and 28 h, respectively). A constant logarithmic increase in cell numbers during culture was observed under both conditions (Figure 1A; *p* < 0.05). On the second day at 39.5 °C, the number of cells increased (twofold) in comparison with that at 37 °C. However, the number of cells after 7 days of culture was similar under both temperatures evaluated. 

### 3.2. Specific Surface Antigens’ Expression, Cell Size, and Granularity Change during Differentiation

Almost 100% of the proliferating B-MSCs were positive for CD90, CD73, and CD44, while about 97% expressed the CD105 antigen. In contrast, hematopoietic stem cell surface markers were detected on the surface of less than 1% of the tested cells (Table 3). These observations indicated that the cells used in this study completely fulfilled the criteria for human B-MSCs [45].

As differentiation progressed, a decrease in the number of cells with B-MSCs-specific markers was detected (Table 3). The most pronounced decrease was observed in cells expressing CD105 (19% of cells at the 28th day) and statistically significant for all days of differentiation (*p* < 0.05). During the differentiation process, a decrease in cell size (signal from forward scatter, FSC) and the content of granularity (signal from side scatter, SSC) was also recorded (Table 3). After 28 days of differentiation, the cells were almost twice as small as B-MSCs and contained about three times less granularity (*p* < 0.05; Table 3). This observation revealed that osteoblasts were smaller in size and had fewer structural granules if compared with their precursors.

The number of HFOB1.19 cells cultured at 37 °C (proliferation conditions) that expressed positive signals for markers specific for B-MSCs was comparable with the number of original B-MSCs. However, the number of CD90-positive cells was slightly lower, as is required for B-MSCs [45], reaching only 91% (*p* < 0.05). This observation indicated that hFOB1.19 cells cultured at 37 °C showed a phenotype similar to that of B-MSCs. Otherwise, cells cultured at 39.5 °C revealed a completely different profile of expressed antigens, characterized by a significant decrease in the number of CD105-positive, CD90-positive, and CD44-positive cells in the analyzed population in comparison with B-MSCs (*p* < 0.05) (Table 3).

### 3.3. B-MSCs’ Differentiation into Osteoblasts Is Associated with Increased Expression of ALPL and SPP1, and the Activity of Alkaline Phosphatase

The expression of *ALPL* increased significantly (23-fold) after 1 day of differentiation as compared with undifferentiating B-MSCs (*p* < 0.05), and such expression was observed until the 28th day of the experiment (Figure 1B). However, the expression of *SPP1* decreased 12-fold on the second day of differentiation in comparison with undifferentiating B-MSCs and then increased gradually until the 28th day, when it finally increased 25-fold in comparison with undifferentiating B-MSCs. On Day 7, the expression of *SPP1* was similar to that of B-MSCs (*p* ≥ 0.05). The observed increase in the expression of *ALPL* and *SPP1* indicated the progressive process of the differentiation of B-MSCs into osteoblasts. 

The hFOB1.19 cells did not show statistically significant differences in the expression of *ALPL* compared with undifferentiated B-MSCs (*p* ≥ 0.05). In contrast, the expression of *SPP1* increased more than 24-fold as compared with the B-MSCs (*p* < 0.05).

**Figure 2:**
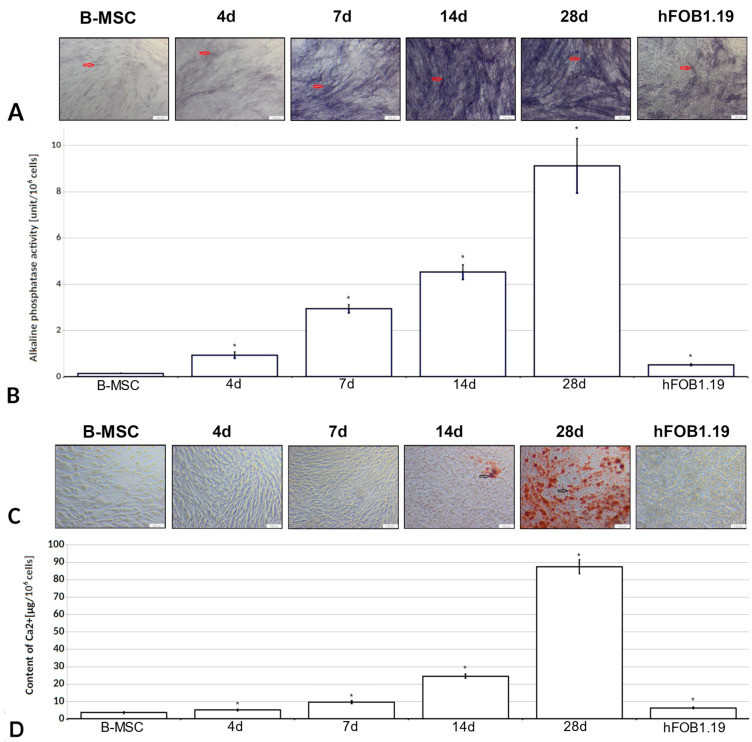
Characteristics of B-MSCs’ differentiation to osteoblasts. B-MSCs were differentiated to osteoblasts for 0, 4, 7, 14, and 28 days. hFOB1.19 cells were cultured for 10 days at 39.5 °C. (**A**) The activity of alkaline phosphatase was visualized by staining with BCIP/NBT (100× magnification; *n* = 2). The red arrows indicate cells with visualized activity of alkaline phosphatase. Bars = 100 µm. (**B**) The activity of alkaline phosphatase was quantified spectrophotometrically with Fast-pNPP (*n* = 5). * The results were considered statistically significant at *p* < 0.05. (**C**) The amount of calcium was detected by staining with Alizarin Red S (100× magnification; *n* = 2). The black arrows indicate calcium deposits in the cell culture. Bars = 100 µm. (**D**) The calcium deposits were quantified spectrophotometrically (562 nm; *n* = 7). * The results were considered statistically significant at *p* < 0.05.

The activity of alkaline phosphatase was detected in all cells tested (Figure 2A) and increased from 0.14 u/10^6^ for undifferentiated B-MSCs to 9 u/10^6^ cells after 28 days of differentiation (Figure 2B). The rate of the increase in the activity of alkaline phosphatase was most pronounced between the fourth and seventh days of differentiation and reached 0.7 u/day per 10^6^ cells) after a steady state (approximately 0.25 u/day). In the case of hFOB1.19 cells, a decrease in the activity of alkaline phosphatase was observed compared with B-MSCs differentiated for 4 days, i.e., 0.5 u/10^6^ cells. All observed changes were statistically significant compared with B-MSCs (*p* < 0.05).

### 3.4. B-MSCs’ Differentiation into Osteoblasts Is Accompanied by Appearance of Mineralization Nodules

The mineralization nodules appeared after 2 weeks and reached the highest concentration on the 28th day of differentiation (87 μg Ca/10^6^ cells) (Figure 2C,D), which was in contrast to undifferentiated B-MSCs (3.5 µg/10^6^ cells). A logarithmic increase in mineralization was observed as differentiation progressed. The average rate of the increase in calcium deposition compared between B-MSCs and cells differentiating for 4 days was approximately 0.5 μg/day (per 10^6^ cells) and increased in differentiating cells (1.5 μg/day at 4–7 days, 2.1 μg/day at 7–14 days of differentiation), reaching a maximum at the last stage of differentiation (4.5 μg/day/10^6^ cells) (*p* < 0.05). On the contrary, hFOB1.19 cells showed similar calcium levels to that found in B-MSCs after 4 days of differentiation (6 μg/10^6^ cells). 

### 3.5. TID Proteins Are Detected in B-MSCs and Osteoblasts

**Figure 3:**
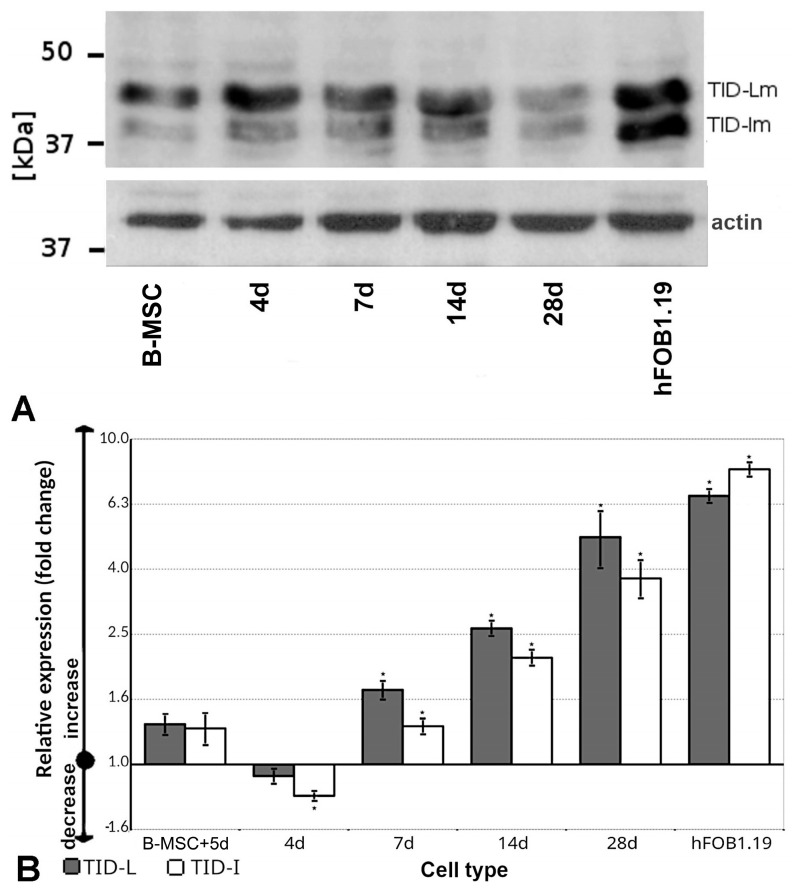
Detection of mRNA and proteins for specific TID isoforms. (**A**) Western blot analysis of TID proteins. Specific TID variants were detected in cell lysates obtained from B-MSCs differentiated for 4, 7, 14, and 28 days and hFOB1.19 cells. For this, 4 μg of protein in the lysate was applied per lane. The membrane was probed with antiactin antibodies to verify the amount of protein applied. All experiments were performed in duplicate. (**B**) The transcription intensity of *TID-L* (black columns) and *TID-I* (white columns) upon differentiation of osteoblasts normalized to undifferentiated B-MSCs (relative expression). The following cells groups were compared with B-MSCs: B-MSCs with an additional 5 days of culture under conditions of proliferation (B-MSC+5d); those at 4, 7, 14, and 28 days of differentiation; and hFOB1.19 (hFOB) cells cultured for 10 days. All experiments were performed in quadruplicate. * The results were considered statistically significant at *p* < 0.05.

TID proteins were detected in all cell types tested with the individual protein isoforms, i.e., the mitochondrial forms (TID-L and TID-I) (Figure 3A). These data showed that each type of cell analyzed, i.e., the B-MSCs, the cells differentiating into osteoblasts, and the hFOB1.19 cells, produced distinct isoforms of this protein.

### 3.6. The Expression of Alternative Splice Variants of TID Fluctuates under Differentiation 

The results of q-PCR indicated the presence of all analyzed variants in the cells tested (the values of ΔCt for *TID-L*, *TID-I*, and *TID-S* for B-MSCs were equal to 4.46, 2.64, and 10.68, respectively). However, the expression of the *TID-S* form was low; thus, it was excluded from further analyses. Notably, the *TID-L* variant predominated over *TID-I* in all cells tested (Figure 3B).

After 4 days of differentiation, the expression of *TID-L* was similar, whereas the expression of *TID-I* decreased in comparison with B-MSCs (*p* < 0.05) (Figure 3B). After 7 days, the expression of both splice variants increased significantly (*p* < 0.05) (Figure 3B) and reached a maximum after 28 days as compared with undifferentiated cells (a 5-fold change for *TID-L* and a 3.7-fold change for *TID-I*). However, we did not find any significant changes in the expression of *TID-L* and *TID-I* after five consecutive days of culturing B-MSCs under conditions of proliferation (*p* > 0.05) (Figure 3B). These observations revealed that the expression of *TID-I* and *TID-L* was stable in B-MSCs but increased with the progression of osteoblasts’ differentiation.

In hFOB1.19 cells, the expression of *TID-L* and *TID-I* increased about 6.7- and 8.1-folds, respectively, in comparison with undifferentiated B-MSCs (*p* < 0.05) (Figure 3B), indicating that the dominant form in hFOB1.19 was the *TID-I* isoform, which was in contrast to the B-MSCs. The expression of both forms was higher in hFOB1.19 cells than in B-MSCs after 28 days of differentiation into osteoblasts. This observation showed that the expression profile of *TID* in hFOB1.19 cells varies from that in B-MSCs.

### 3.7. Silencing the TID Splice Variants Induces the Expression of SPP1 

A standardization procedure showed that the efficiency of *TID* silencing varied and was dependent on the day of transfection, the type of siRNA, and the cell types used (Table S1, Supplementary Materials). In this study, DNAJA3_8 was used because the optimization data showed it to be the most efficient silencer. 

**Figure 4:**
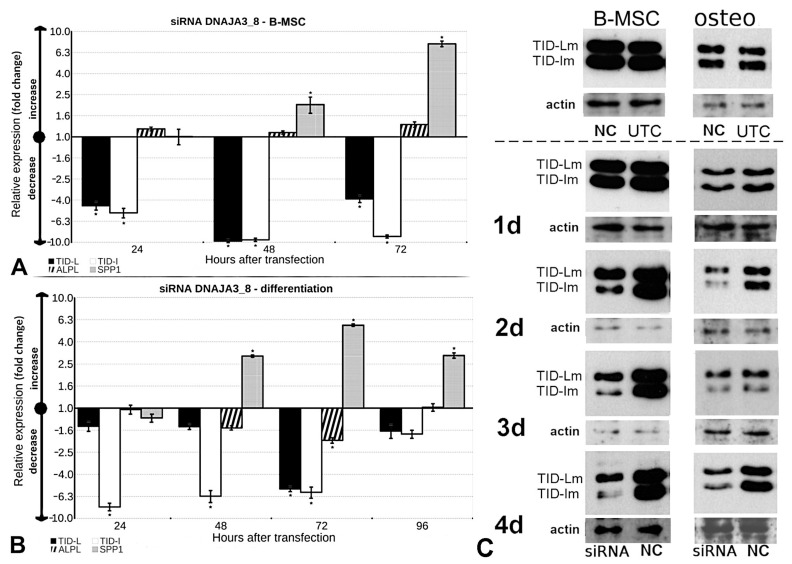
Silencing of the *TID* gene. (**A**) Effect of siRNA_DNAJA3_8 transfection on the expression of the *TID-L* (black columns) and *TID-I* (white columns) isoforms, alkaline phosphatase (*ALPL*; black diagonal stripes), and osteopontin (*SPP1*; gray grid) in B-MSCs. The gene expression of the silenced cells was compared with the negative control’s expression (treated with AllStar Negative Control siRNA on the corresponding day of proliferation or differentiation). Two independent experiments and four q-PCR analyses were performed. * The results were considered statistically significant at *p* < 0.05. (**B**) The influence of siRNA_DNAJA3_8 transfection on the expression of the *TID-L* and *TID-I* isoforms, alkaline phosphatase (*ALPL*), and osteopontin (*SPP1*) in cells differentiated to osteoblasts. Gene expression in the silenced cells was compared with the negative control’s expression (AllStar Negative Control siRNA). * The results were considered statistically significant at *p* < 0.05. (**C**) Western blot analysis of the effect of silencing the expression of *TID* on TID-L and TID-I protein. B-MSCs undifferentiated and differentiated into osteoblasts (osteo) were cultured for 1 (1d), 2 (2d), 3 (3d), or 4 (4d) days after transfection with siRNA. The intensity of the signal from TID proteins in cells transfected with DNAJA3_8 siRNA (designated as siRNA) was compared with the intensity of the signal for the negative control (NC; bottom panel). To demonstrate that the transfection method had no effect on the amount of TID proteins, the negative control (AllStar Negative Control siRNA) was compared with untransfected cells (UTC; top panel). Two independent experiments were performed. m, mitochondrial form of TID proteins.

Directly after silencing, the expression of *TID-L* and *TID-I* was significantly reduced, but less so for *TID-L* in cells differentiating into osteoblasts as compared with B-MSCs (Figure 4A,B). Optimal inhibition of the synthesis of TID protein was observed 3 days after the transfection (Figure 4C). For both the *TID-L* and *TID-I* splice variants, a meaningful effect of silencing was achieved on each day of analyzing the B-MSCs as compared with the negative control (*p* < 0.05), whereas, in cells differentiating into osteoblasts, the effect on *TID-L* was observed only at the third day after silencing and that for *TID-I* was observed during 3 days of culture (*p* < 0.05). Our observations showed that the siRNAs used did not affect the expression of both *TID* forms to the same extent.

The silencing procedure had also some effects on the expression of the *ALPL* and *SPP1* genes. In the case of *ALPL*, a twofold decrease was detected exclusively in differentiating cells 72 h after transfection (*p* < 0.05). In the case of *SPP1*, except for the first day after transfection, a significant increase in mRNA in both proliferating and differentiating cells with maximum expression at the third day after transfection was noted (a 7.6-fold increase compared with the negative control for B-MSCs and a 5.6-fold increase in comparison with the negative control for cells differentiating into osteoblasts) (*p* < 0.05) (Figure 4A,B). These results suggest a possible link between the silencing of the *TID* gene and the expression of *SPP1*.

Silencing the expression of *TID* had no impact on cell proliferation (*p* ≥ 0.05). There were also no statistically significant differences in the activity of alkaline phosphatase (*p* ≥ 0.05) and the amount of the calcium deposits (*p* ≥ 0.05) in the cell cultures after silencing (Table 4).

## 4. Discussion

TID proteins have been previously shown to be involved in key pathways of stem cells’ differentiation into osteoblasts [21]. However, the effect of specific *TID* splice variants on osteogenic processes has not been clarified to date. Therefore, we analyzed the expression profiles and protein products of the *TID* variants (*TID-L* and *TID-I*) in B-MSCs during proliferation and differentiation into osteoblasts. 

To standardize the study conditions, a medium free of serum and other animal components was used, which enabled the cells to retain their phenotype, morphology, and ability to differentiate. Under such conditions, the cells revealed greater viability and proliferative capacity compared with cells cultured in the standard media supplemented with animal serum [50]. In contrast, serum-free medium can be used for transplantation, analogous to those supplemented with an autologous platelet lysate [51].

In this study, the B-MSCs were isolated and differentiated into osteoblasts under in vitro conditions. While the proliferation of B-MSCs was constant, the proliferation rate of differentiating cells increased at the beginning of the study and then decreased. This effect is consistent with observations from other studies and related to the formation of a multilayered cell surface at the onset of osteoblastogenesis [52]. It was demonstrated that a high cell density allows acceleration of the differentiation and expression of osteogenic genes [53].

The International Society for Cellular Therapy has proposed minimum criteria for B-MSCs [45]. These criteria are mainly based on stable expression of cell surface markers, i.e.,CD73, CD90, and CD105, independent of the number of cell passages [2,8]. The analyses showed that the stem cells tested in the current study expressed these antigens on their surface. We observed a slight decrease in the number of cells with the expression of endoglin (CD105 antigen) after two additional passages (but without statistical significance). In the literature, however, a decrease not only in the CD105 [54] but also the CD73 antigens was reported [55]. Finally, the expression of all markers specific for the B-MSC phenotype decreased upon differentiation into osteoblasts, which was also confirmed in previous studies [55,56,57]. The majority of the B-MSC population in our study lost the expression of endoglin on their surface upon differentiation into osteoblasts. Endoglin is a coreceptor that interacts with Type I and Type II TGFβ and BMP receptors [12,58]. Populations with high levels of CD105-positive cells showed increased expression and secretion of TGF-β1, but it has been shown that an elevated concentration of this growth factor can induce the suppression of osteogenesis. Hence, cells presenting low levels of endoglin have greater potential for osteoblasts’ differentiation, and silencing of the expression of endoglin led to increased expression of the genes associated with early osteogenesis [59]. Nevertheless, studies of peripheral ligament cells have shown that endoglin is required for BMP-2-induced osteoblastogenesis [12]. It is likely that endoglin fulfills pleiotropic functions in osteogenesis, depending on the differentiation stage and the type of cells. Nevertheless, we demonstrated here that endoglin appears to be a useful marker of B-MSCs. 

The process of differentiation of B-MSCs into osteoblasts in our study was also monitored by analyses of other commonly used markers, i.e., the activity of alkaline phosphatase, calcium deposition, and the expression of *SPP1* and *ALPL1*. The data clearly showed efficient differentiation of the B-MSCs into osteoblasts, similar to previously published reports [53,60,61,62].

As control cells, immortalized human fetal osteoblasts (hFOB1.19) were used here, which, analogous to B-MSCs, can be differentiated into other cell types, such as adipocytes, chondrocytes, and mature osteoblasts [41]. This cell line has been transfected with the thermosensitive large T antigen of SV40, so that the decrease in cell proliferation slows down at 39.5 °C, while the expression of osteoblastic markers increases [40]. Thus, the hFOB 1.19 cells are recommended as the most appropriate and, so far, the only available reference cells for the differentiation of B-MSCs into osteoblasts. Our data showed slightly decreased activity of alkaline phosphatase in this cell line, which was in contrast to other studies [44]. However, we did not add any supplements to the medium, which may have accelerated differentiation, e.g., exosomes with lncRNA from umbilical cord mesenchymal stem cells [63]. In addition, a low amount of hydroxyapatite containing calcium ions has been confirmed by others [43]. The number of cells expressing CD44, CD73, CD90, and CD105 surface antigens was similar to that of B-MSCs exclusively when the hFOB1.19 cells were cultured at a lower temperature (37 °C). The presence of these antigens on hFOB1.19 has been demonstrated previously [41,42]. The present study showed that hFOB1.19 cells showed the typical phenotype of the initial stage of osteogenesis and were correctly classified as progenitor cells, such as pre-osteoblasts, but not as mature osteoblasts. Thus, the increased expression of *SPP1* by these cells in our study seems to be unexpectedly surprising. 

We detected different variants of the TID proteins in B-MSCs under conditions of both proliferation and differentiation. More importantly, the expression of transcript variants of *TID* (*TID-L* and *TID-I*) increased significantly upon differentiation. Kulterer et al. (2007) examined oligonucleotide microarrays by altering the expression of nearly 30,000 transcripts during the differentiation of mesenchymal stem cells into osteoblasts, but the change in the expression of *TID* during differentiation into osteoblasts was less than twofold compared with B-MSCs [2]. In our study, convergent results were obtained for the first 7 days of B-MSCs’ differentiation, followed by an increase of more than twofold in the expression of *TID-L* and *TID-I*. The discrepancies observed between the study of Kulterer et al. and our observations may result from the differences in the methodology and the composition of the media used. The increase in expression the of *TID-L* and *TID-I* during differentiation can be related to the increase in the mitochondrial DNA copy number after 7 days of differentiation [64]. Simultaneously, long-term silencing of the expression of *TID* reduced the number of mitochondrial DNA copies [65]. Similarly, blocking TID in mouse heart muscle reduced copies of mitochondrial DNA by decreasing the level of POLG polymerase [66]. Furthermore, the carboxyl end of a protein homologous to TID (Mdj1) was responsible for binding to the mitochondrial nucleoid in yeast [67]. However, TID can also affect mitochondrial function by decreasing the expression of mitochondrial respiratory complex proteins, membrane potential, and overall mitochondrial mass [68]. Although only a qualitative protein analysis was performed in our study, the protein level measured by Western blotting appeared to be slightly different from the mRNA level, which was particularly evident after 28 days of differentiation. Since the proteins can be degraded, their levels may not correlate with the mRNA expression. We also cannot exclude that the expression of the *TID* gene increased before the 4th and 28th days of differentiation.

On the first day after transfection, silencing of the expression products of *TID* was effective at the transcript, but not protein, level. Considering that the *TID-L* and *TID-I* proteins are stable in the cell for hours, this observation may have resulted from the presence of endogenous protein that has not yet been degraded [23].

As measured by the activity of alkaline phosphatase and mineralization nodules, silencing the *TID* gene did not affect the proliferation of B-MSCs and their differentiation into osteoblasts; however, silencing of *TID* transcripts resulted in increased expression of *SPP1* (osteopontin) in both proliferating as well as differentiating conditions. These results may suggest that TID affects the expression of *SPP1*. Moreover, a functional relationship can be proposed to explain these results, because NFκB is a positive regulator of the expression of *SPP1* [69]. Otherwise, TID is a negative regulator of NFκB’s activity; thus, silencing of *TID* transcripts can affect the translocation of NFκB to the cell nucleus, where it induces the expression of various genes [70], including *SPP1.* This phenomenon may explain the increase in the expression of *SPP1*, even in undifferentiated stem cells. 

Our preliminary study demonstrated, for the first time, that the expression of *TID-L* and *TID-I* changes under the differentiation of B-MSCs into osteoblasts and may influence the expression of *SPP1*. However, for a better understanding the mechanisms of the processes associated with TID’s effect during osteogenic differentiation of B-MSCs, further research with mesenchymal cells from other tissues, e.g., cord blood, adipose tissue, dental pulp, and amniotic fluid, as well as the involvement of overexpression and complete knockout study models are needed. Such an approach would enable us to clarify the mitochondrial role of TID in the differentiation of B-MSCs into osteoblasts and the direct functional link between TID and osteopontin. If approved, the effect would be further exploited to treat diseases where the expression of *SPP1* is deficient, such as ectopic calcification in blood vessels, the heart, and kidneys or microcalcification in early-stage breast cancer. Osteopontin inhibits the formation of hydroxyapatite crystals in blood vessels [71], so siRNA that silences the expression of *TID* and increases the expression of *SPP1* would be delivered via lipid or polymer nanoparticles by direct injection. The efficiency of the therapy could be improved by the addition of a protein or antibody specific for the target tissue. Such types of drugs have already been approved by medical organizations [72]. 

## 5. Conclusions

In summary, during the differentiation of B-MSCs into osteoblasts, the expression of the *TID-L* and *TID-I* splice variants was significantly elevated. The dominant splice variant detected in B-MSCs and cells differentiating into osteoblasts was *TID-L*, whereas *TID-I* was mainly present in the hFBO1.19 cell line. Silencing of transcription variants of *TID* did not affect the activity of alkaline phosphatase, mineralization, or cell proliferation, but induced the expression of *SPP1*. Shifts in *TID-I* and *TID-L* levels most likely allow the maintenance of normal homeostasis in cell culture, but their specific effects on the initiation of osteogenesis need further investigation.

## Figures and Tables

**Table 1:** Culture times used in the study assays.

Cells	B-MSCs		hFBO1.19	
Experimental Conditions	Proliferation (days)	Differentiation (days)	37 °C (days)	39.5 °C (days)
Proliferation assay (Alamar Blue)	1, 2, 3, 5	1, 2, 3, 4, 5, 7, 14, 28	1, 2, 3, 7	1, 2, 3, 7
Flow Cytometry	5	4, 7, 14, 28	10	10
Alizarin red, alkaline phosphatase activity, Western blotting, RT q-PCR	5	4, 7, 14, 28	-	10
siRNA	1, 2, 3	1, 2, 3, 4, 5, 14	-	-

**Table 2:** Sequences of primers used in the study, and the size of the products.

Transcript	Name	Sequence	Primer Length (bps)	Product Length (bps)
*GAPDH* ^1^	GAPDH_f	5′-GAG TCA ACG GAT TTG GTC GTA-3′	21	245
	GAPDH_r	5′-GCC CCA CTT GAT TTT GGA G-3′	19	245
*HPRT*1 ^2^	HPRT_s	5′-TGA CAC TGG CAA AAC AAT GCA-3′	21	94
	HPRT_a	5′-GGT CCT TTT CAC CAG CAA GCT-3′	21	94
*SPP1* ^1^	SSP1_F	5′-GCA ACC GAA GTT TTC ACT CC-3′	20	339
	SPP1_R	5′-GCT CTC ATC ATT GGC TTT CC-3′	20	339
*ALPL* ^1^	ALPL_F	5′-CAA GCA CTC CCA CTT CAT CTG-3′	21	203
	ALPL_R	5′-CCA GCA AGA AGA AGC CTT TG-3′	20	203
*TID-S* ^2^	P3f	5′-CAG CCT CAG GAA GAA ACC ATC-3′	21	399
	P3r	5′-GGG ATC GTC ACG TTG ATC GTC-3′	21	399
*TID-I* ^1&2^	P1f	5′-CTG GGA TAT CAT GAG GTA AAC-3′	21	716
	P2r	5′-CCA GTG GAT CTT TTT CCA GAG-3′	21	716
*TID-L* ^2^	P1f	5′-GTT GAC ATT CAA TCA AGC TGC-3′	21	821
	P1r	5′-CTG GGA TAT CAT GAG GTA AAC-3′	21	821
*TID-L* ^1^	TL_F	5′-TCA CCG TGA ACA TCA TGG AC-3′	20	733
	TL_R	5′-GAA AGG AAT CCC TCC TCG TC-3′	20	733

^1^ Primers used to analyze the expression silencing of *TID* isoforms and the expression of osteogenic markers. ^2^ Primers used to study the expression of *TID* after 4, 7, 14, and 28 days of B-MSCs’ differentiation into osteoblasts.

**Table 3:** Phenotypic evaluation of cell types upon osteoblastic differentiation.

Cell Type		% of Cells with the Antigen in the Population								Value Compared with B-MSCs (%)	
		CD105	CD90	CD73	CD90 + CD105	CD105 + CD73	CD73 + CD90	CD44	HSC Mark.	FSC	SSC
B-MSCs		97	100	100	98	99	100	99	1	100	100
B-MSCs + 2 passages		93	100	100	94	95	99	99	0	129	137
Days of differentiation	4	87 *	98	99	82 *	87 *	97	99	0	82	74
	7	80 *	97 *	99	77 *	82 *	97	99	0	83	63
	14	56 *	97	99	54 *	56	95	99	0	65	44
	28	19 *	78	86	15 *	25 *	75	87	0	52 *	29 *
hFOB1.19 @37 °C		95	91 *	99	88 *	95	91	99	0	113	32 *
hFOB1.19 @39.5 °C		63 *	28 *	96	1 *	34 *	12 *	91 *	0	79	60

CD73 (APC), CD90 (FITC), and CD105 (PerCP-Cy5.5) are B-MSC-specific markers. HSC corresponds to the set of markers specific for hematopoietic stem cells, which included CD34, CD11b, CD19, CD45, and HLA-DR (all conjugated to PE). The B-MSCs were differentiated to osteoblasts for 4, 7, 14, and 28 days and cultured for two additional passages under conditions of proliferation (B-MSCs + 2 passages). The hFOB1.19 cells were cultured at 39.5 °C (@39.5 °C) and at 37 °C (@37 °C) for 10 days. Cells were analyzed in triplicate (*n* = 3) using a flow cytometer (FACS Aria). Histograms are shown in the Supplementary Materials (Figure S1). Assessment of the cells’ size (FSC) and granularity (SSC) upon a change in phenotype from undifferentiated to osteoblastic stage was also performed, expressed as a percentage of B-MSCs. * The results were considered statistically significant at *p* < 0.05.

**Table 4:** Effect of silencing the expression of the *TID* gene on selected markers of osteogenesis.

Cell Type		Relative Proliferation	Relative Activity of Alkaline Phosphatase	Relative Number of Calcium Deposits
B-MSCs		99.7 ± 1.6%	102.5 ± 2.2%	-
Day of Differentiation	3rd	97.7 ± 1.0%	103.5 ± 4.8%	-
	5th	99.3 ± 0.9%	100.3 ± 2.6%	-
	14th	-	-	100.6 ± 4.5%

Proliferation rate, activity of alkaline phosphatase, and number of calcium deposits after silencing of the *TID* gene compared with the negative control (AllStar Negative Control siRNA) in B-MSCs on selected days of differentiation.

## Data Availability

The original contributions presented in the study are included in the article/Supplementary Material. Further inquiries can be directed to the corresponding authors.

## Supplementary Materials

The following supporting information can be downloaded at https://www.mdpi.com/article/10.3390/cells13121021/s1. Table S1 Efficiency of silencing various forms of siRNA against TID transcripts. Figure S1. Examples of histograms from the cytometric analysis.
