# Supplementary material for: Analysis of the TID-I and TID-L Splice Variants’ Expression Profile under In Vitro Differentiation of Human Mesenchymal Bone Marrow Cells into Osteoblasts
Source: Cells. 2024 Jun 11;13(12):1021. doi: 10.3390/cells13121021 (PMC11201664; doi:10.3390/cells13121021)
Supplement: Supplementary file 1 [file cells-13-01021-s001.zip › cells-3042130-supplementary.pdf]

Supplementary Materials

Analysis of the *TID-I* and *TID-L* Splice Variants' Expression Profile under In Vitro Differentiation of Human Mesenchymal Bone Marrow Cells into Osteoblasts

Daniel Krakowian, Marta Lesiak, Aleksandra Auguściak-Duma, Joanna Witecka, Damian Kusz, Aleksander L. Sieroń, Katarzyna Gawron

1. Formulas

I. The population doubling time:

$$\mathbf{T_D = \frac{log(2) \times (t_1 - t_0)}{log(N_1) - log(N_0)}}$$	TD - population doubling time (in hours)<br>t_0 - time of seeding (0 hours),<br>t_1 - end of culture (72 hours after seeding),<br>N_0 - absolute number of cells seeded per well<br>N_1 - absolute number of cells at the end of the culture (in the same wells as N_0)

II. Standard curve for calculating the amount of calcium in cell culture:

$$\mathbf{Ca\ [\mu g] = \frac{A}{0.47}}$$	A - the absorbance value<br>Ca - the amount of calcium in µg

III. Standard curve for calculating the activity of alkaline phosphatase in cell culture:

$$\mathbf{ALP\ [u] = \frac{A}{19.4}}$$	A - the absorbance value<br>ALP - units of alkaline phosphatase activity

IV. Standard curve for calculating the protein concentration in cell culture:

$$\mathbf{BSA\ [mg/mL] = \frac{A}{1.23}}$$	A - the absorbance value<br>BSA - concentration of bovine serum albumine

V. The efficiency of PCR reactions for each pair of primers:

$$\mathbf{E = \left(10^{\frac{-1}{tg\alpha}}\right) - 1}$$	E - efficiency in %<br>α - angle formed between the OX axis and the curve for the Ct function from the tenfold dilution (Ct - temperature at which the obtained fluorescence signal exceeds the background value)

VI. ΔCt - Gene expression compared to housekeeping gene:

$$\mathbf{\Delta Ct = Ct_b - Ct_k}$$	Ct - cycle number when fluorescence is above threshold<br>b - tested gene<br>k - housekeeping (constitutive) gene

VII. $2^{-\Delta\Delta Ct}$ - Gene expression compared to bone marrow mesenchymal stromal cells (B-MSC)

$$\mathbf{2^{-\Delta\Delta Ct} = \frac{(2^{-\Delta Ct})_X}{(2^{-\Delta Ct})_{B\text{-}MSCs}}}$$	$2^{-\Delta Ct}$ - number of copies of the tested gene per one copy of the constitutive (housekeeping) gene<br>X - cells on a given day of differentiation<br>B-MSCs - bone marrow mesenchymal stromal cells

2. siRNA sequences and efficiency of silencing

Table S1. Efficiency of silencing various forms of siRNA against *TID* transcripts.

siRNA (name and sequence)	Day after transfection	Proliferation		Differentiation	
		TID-L	TID-I	TID-L	TID-I
DNAJA3_5: CTC CGG CAT GGA AAC CAT CAA	1	0.31	0.3	0.94	0.21
	2	0.45	0.59	0.46	0.54
	3	0.31	0.37	0.62	1.19
	4	-	-	11.1	1.03
DNAJA3_7: CCC GAG CGC TGC TGA CAT TGA	1	0.2	0.18	0.67	0.7
	2	0.14	0.19	0.55	0.6
	3	0.14	0.19	0.56	0.19
	4	-	-	0.61	0.52
DNAJA3_8: CCG GAT TAA CAG CTA CGG CTA	1	0.22	0.18	0.68	0.13
	2	0.1	0.1	0.68	0.16
	3	0.25	0.11	0.18	0.17
	4	-	-	0.62	0.58
DNAJA3_9: AAA GGC CAT GCT TAC AGC TTA	1	0.24	0.18	0.74	0.23
	2	0.11	0.14	0.32	0.13
	3	0.15	0.17	0.58	0.19
	4	-	-	0.47	0.74

The table shows the $2^{-\Delta\Delta Ct}$ value, expressed as the ratio of 2-ΔCt for cells transfected with a given siRNA (DNAJA3_5, DNAJA3_7, DNAJA3_8, DNAJA3_9) and $2^{-\Delta Ct}$ for cells transfected with a negative control (All Star Negative Control siRNA).

3. Flow cytometry-based phenotypic assessment of cells

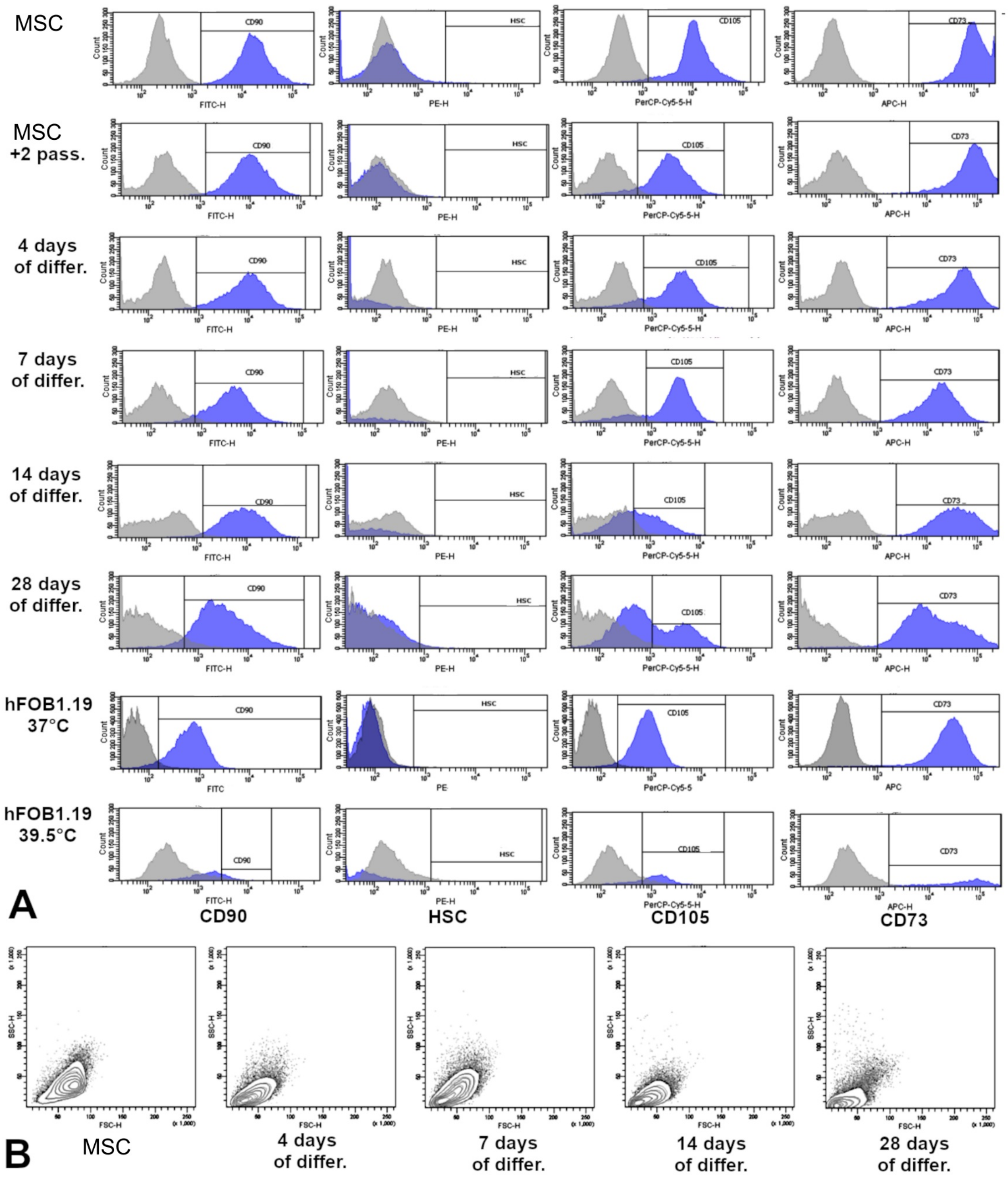

Figure S1. Example histograms from cytometry analysis.

B-MSC and hFOB1.19 were analysed by flow cytometry under proliferation and differentiation conditions. A - Histograms from one of three replicate analyses. The phenotype of the cells was checked for the presence of surface markers of mesenchymal bone marrow cells (CD90, CD105, CD73) and markers of haematopoietic stem cells (HSC); blue filling is cells with given antigen, grey filling is cells treated with isotype control (an image of two independent samples are superimposed). B - Changes in size (FSC-H axis) and granularity (SSC-H axis) during differentiation of B_MSCs into osteoblasts. The cytometry setting was the same at each time point.
